# Unveiling the Silent Crisis Amidst Tackling Hepatitis B in African Prisons ‐ A Public Health Emergency

**DOI:** 10.1002/hsr2.70543

**Published:** 2025-03-10

**Authors:** Godfred Yawson Scott, Abdullahi Tunde Aborode, Ridwan Olamilekan Adesola, Chime Onyinye Hope, Olayide Dipeolu, Favour Akinfemi Ajibade, Abraham Olutumininu Akiyode, Stephen Tetteh Engmann, Abdullahi Jamiu, Awoyemi Praise‐God Adetunji, Joseph Agyapong, Toluwalope Yinka Oni, Mpanga Derick Denis, Isreal Onifade Ayobami, Adetolase Azizat Bakre

**Affiliations:** ^1^ Department of Medical Diagnostics Kwame Nkrumah University of Science and Technology Kumasi Ghana; ^2^ Department of Research and Development Healthy Africans Platform Ibadan Nigeria; ^3^ Department of Veterinary Medicine, Faculty of Veterinary Medicine University of Ibadan Ibadan Nigeria; ^4^ Department of Community Medicine College of Medicine, Enugu State University of Science and Technology Enugu Nigeria; ^5^ Department of Biology University of Texas Permian Basin Odessa Texas USA; ^6^ Faculty of Veterinary Medicine University of Ibadan Ibadan Nigeria; ^7^ Family Medicine Unit, Manna Mission Hospital Accra Ghana; ^8^ Department of Dietetics University of Ghana Accra Ghana; ^9^ Department of Biomedical Sciences and Pathobiology Virginia Tech Blacksburg Virginia USA; ^10^ Department of Biochemistry Federal University of Technology Akure Gaga Nigeria; ^11^ Africa Medical and Behavioral Sciences Organization‐ Research Arm Kampala Uganda; ^12^ Department of Biology University at Albany Albany New York USA

**Keywords:** Africa, hepatitis B, prison, public health

## Abstract

**Background:**

Hepatitis B is one of the major global health issues, which presents a particularly severe challenge within the confines of African prisons, characterized by high rates of transmission and limited access to adequate healthcare. The prevalence of Hepatitis B in these settings represents a silent crisis.

**Objective:**

This research highlights the critical public health emergency posed by Hepatitis B in African prisons, underscoring the need for urgent intervention and comprehensive strategies.

**Methods:**

A comprehensive literature search was conducted to investigate the public health challenges posed by Hepatitis B in African prisons. The search focused on peer‐reviewed articles, policy documents, and original literature published from 2000 to 2024. Databases including PubMed, Scopus, Web of Science, and Google Scholar were utilized.

**Results:**

The prison environment, marked by overcrowding, inadequate sanitation, and high‐risk behaviors, fosters the rapid spread of Hepatitis B. The transmission is further exacerbated by limited access to vaccination, insufficient screening programs, and a lack of awareness among inmates and prison staff. Consequently, the incidence of Hepatitis B in African prisons is significantly higher than in the general population, creating a reservoir of infection that poses a broader public health threat upon prisoners' release. Addressing this crisis requires a multifaceted approach.

**Conclusion:**

This research calls for immediate and sustained action to mitigate the Hepatitis B crisis in African prisons. By prioritizing this issue within public health agendas, we can reduce transmission rates, improve health outcomes for inmates, and protect broader community health. The urgency of addressing Hepatitis B in African prisons cannot be overstated, as it represents a critical juncture in the fight against infectious diseases in marginalized populations.

## 1 Introduction

Hepatitis B, a potentially life‐threatening liver infection caused by the hepatitis B virus (HBV), is a global health concern with significantly higher incidence rates in the incarcerated populations of African prisons [1]. High‐contagious HBV is spread by direct and indirect contact with contaminated blood and bodily fluids (such as semen and vaginal discharge). It can cause acute or chronic necro‐inflammatory liver disorders [2]. Even though the majority of people have access to an effective vaccine, the high prevalence of chronic liver disease morbidity and mortality caused by the hepatitis B virus now poses a significant global public health concern [2]. Chronic hepatitis caused by HBV affects over 360 million individuals globally and accounts for 620,000 annual deaths [2]. The congested living conditions, lack of adequate healthcare facilities, and limited access to preventive measures contribute to the rapid spread of this infectious disease among prisoners [2]. However, the issue remains underreported and underrepresented in public health discussions, making it a silent epidemic that demands immediate attention and action.

There have been three identified zones of chronic HBV infection prevalence: high (8.0%), intermediate (2%–8%), and low (2% or lower). Hepatitis B surface antigen (HBsAg) seropositivity is greater than 8% in high‐endemic areas, where 45.0% of the world's population resides [3, 4, 5]. Among other regions, these include the Middle East, Southeast Asia, the Pacific Basin, and a few Eastern European nations [1, 5]. Sub‐Saharan Africa has the most excellent endemicity rate in the world, with over 8% of the population infected with the virus and almost 50 million chronic carriers, mostly in hyperendemic nations like Nigeria, Namibia, Gabon, Cameroon, and Burkina Faso [6], while some of the epidemiological studies have been summarized in Table 1 below. The northern countries of the continent, including Egypt, Tunisia, Algeria, and Morocco, are categorized as having moderate levels of endemicity, ranging from 2% to 7% [7]. On the other hand, hepatitis B virus carriers make up less than 2% of the populations in North America, Western and Northern Europe, and certain regions of South America and Australia [7]. The prevalence of hepatitis B virus infections in the general public, as well as important communities like prisons, however, is barely documented in Africa.

**Table 1:** Summary of epidemiological studies on hepatitis B in African prisons.

Country	Key findings	Recommendations	References
Nigeria	15% prevalence of Hepatitis B among inmates; low vaccination coverage (5%).	Implement routine screening and vaccination programs in prisons.	[3]
Ghana	22% co‐infection of HBV with HIV; increased risk among male inmates.	Integrate HBV management with existing HIV programs in prisons.	[4]
Kenya	Sharing razors and unprotected sexual activities identified as major risk factors.	Educate inmates on risk reduction and improve access to personal hygiene products.	[5]
South Africa	HBV underreported in prison health data; limited epidemiological surveillance.	Establish surveillance systems and standardized reporting mechanisms.	[6]
Sudan	There are around 30% prevalence among newly incarcerated individuals; poor healthcare access.	Increase funding for prison healthcare and prioritize HBV testing at intake.	[7]
Uganda	Higher transmission rates observed during prolonged incarceration.	Develop comprehensive preventive care strategies targeting long‐term inmates.	[8]

The Table 1 above provides an overview of epidemiological studies focusing on Hepatitis B in African prisons, summarizing their findings and recommended actions to address the crisis effectively. However, regular social and physical interactions between the inmates and their officials occur in prison, and the typical instances of these encounters include intravenous drug use [8]. As a result, compared to the general population, blood‐borne illnesses like HBV are typically more common among prison populations [9]. It is also commonly recognized that inadequate infection control and prevention (IPC) systems and overcrowding are major problems in many prisons. Considering the high probability of infection‐risk behavior engagements among prisoners and the generally poor environmental conditions, it is possible that the prison environment could act as a breeding and spreading ground for infectious disease pathogens, of which HBV is important. The significance of this current review stems from the fact that the inmates are merely serving jail sentences before being freed. It might, therefore, have catastrophic effects on a country's public health system if infected prisoners who are freed from prison act as reservoirs for the virus.

The current review acknowledges that the implications of HBV epidemic within prison walls extend far beyond the health of incarcerated individuals. Upon release, affected individuals or prisoners carry the virus back into their communities, exacerbating the spread of the disease and imposing additional strains on already burdened public health systems. The stigma associated with both incarceration and infectious diseases further complicates the reintegration process, leading to cycles of poverty, marginalization, and continued health disparities. This initiative highlights the interconnectedness of prison health with public health, advocating for interventions that recognize and address the continuity of care from incarceration to community re‐entry. Again, this review sheds on evidence‐based policies and programs that can effectively reduce the incidence of Hepatitis B within prisons, support affected individuals during and after their release, and raise awareness about the silent epidemic of Hepatitis B in prisons.

### 1.1 Methods of Literature Search

A comprehensive literature search was conducted to investigate the public health challenges posed by Hepatitis B in African prisons. The search focused on peer‐reviewed articles, policy documents, and gray literature published from 2000 to 2024. Databases including PubMed, Scopus, Web of Science, and Google Scholar were utilized. Keywords and Boolean operators such as “Hepatitis B” AND “prisons” AND “Africa”, “Hepatitis B transmission” AND “prison populations”, “public health” AND “African correctional facilities” were applied. Search results were screened for relevance, emphasizing studies addressing epidemiology, risk factors, health system challenges, and interventions for Hepatitis B in African prison settings. Inclusion criteria focused on studies presenting data on prevalence, health disparities, or policy implications. Reports from organizations such as the World Health Organization (WHO), Centers for Disease Control and Prevention (CDC), and African Union (AU) were also reviewed to incorporate global and regional perspectives. Relevant citations from key articles were examined to ensure a robust and exhaustive review of the topic.

## 2 Transmission Dynamics of Hepatitis B Infection in African Settings

### 2.1 Modes of Hepatitis B Transmission in Prisons

HBV is a significant concern in prison settings due to the increased risk of transmission among inmates and correctional workers [10, 11]. The prevalence of hepatitis B and C virus infections, as well as the human immunodeficiency virus (HIV), is significantly higher among prisoners worldwide compared to the general population [12]. Hepatitis B can be transmitted in prisons through various modes. In correctional facilities, the risk of HBV transmission is heightened, and immunization for hepatitis B is recommended for all adults in these settings [13, 14].

Potential means of HBV transmission in prisons include sharing contaminated needles used for drug injections, sharing toothbrushes, tattooing, body piercings, and high‐risk sexual behavior [15, 16, 17, 18]. Additionally, ongoing transmission of hepatitis B virus infection among inmates at a state correctional facility has been reported, suggesting that close contact and exposure to infected bodily fluids may contribute to transmission within prison settings [19, 20].

### 2.2 Factors Contributing to the Hidden Epidemic

The increased transmission of HBV and other infections among inmates is caused by several environmental and societal factors variables [21]. The primary factor associated with Hepatitis B (HBV) infection in prisons in Africa is the geographical region of origin of the inmates. A study conducted in Switzerland's largest pretrial prison found that the prevalence of HBV infection was significantly associated with the region of origin of the participants [11]. Inmates from sub‐Saharan Africa had a higher prevalence of chronic HBV infection compared to those from the other areas [11]. In addition to contributing risk factors like birthplace, older age, and having sexual partners with a history of hepatitis, homelessness may potentially be a risk factor for HBV infection [22]. The literature is less sure of the association between hepatitis B and age [11]. In sub‐Saharan Africa, incarceration at a younger age is associated with HBV infection [19, 23].

African societal and cultural behaviors significantly influence the transmission and management of Hepatitis B in prison settings. In many African regions, communal living and close‐knit social structures often extend into prison environments, where overcrowding amplifies these dynamics [24]. High levels of physical proximity and resource sharing, coupled with limited awareness about Hepatitis B transmission, contribute to an elevated risk of spread. Furthermore, stigmatization of Hepatitis B as a “taboo disease” in some African cultures discourages testing, disclosure, and treatment adherence among incarcerated individuals, perpetuating the silent crisis.

African prisons are often characterized by inadequate healthcare services compared to prisons in other regions, which exacerbates the burden of HBV [13, 15]. Limited availability of diagnostic tools, vaccines, and antiviral treatments in correctional facilities reflects broader systemic health challenges on the continent. These issues are compounded by insufficient funding and healthcare worker shortages. Unlike many developed regions where prison health aligns with public health policies, African prisons are frequently isolated from national healthcare systems, leaving inmates with inadequate preventive or curative care for Hepatitis B.

Behavioral practices within African prisons differ from those in other regions and increase vulnerability to Hepatitis B [22]. Tattooing with unsterilized tools, sharing of personal hygiene items like razors, and illicit drug use are prevalent in many prisons and facilitate transmission of bloodborne infections [22]. Additionally, sexual violence and unprotected consensual sexual activities, often ignored or underreported, heighten the risk of infection. In some African cultures, perceptions of prison as a place of isolation rather than rehabilitation contribute to a lack of public concern about improving health practices in these settings.

Unlike many other regions, African countries often lack robust policies targeting HBV prevention and treatment in prisons. There is minimal advocacy for routine screening or vaccination of inmates, a standard practice in developed regions [23]. Moreover, weak enforcement of international prison health guidelines and limited political will to prioritize the health of incarcerated populations further differentiate Africa from other regions. This neglect stems from broader societal attitudes toward prisoners, viewing them as undeserving of healthcare resources, thereby perpetuating the cycle of neglect and disease transmission as shown in Table 2 below.

**Table 2:** Factors contributing to the hepatitis B Crisis in African prisons.

Category	Factors	Impact of hepatitis B crisis
Overcrowding and living conditions	1. Overcrowding in prison cells 2. Poor sanitation and hygiene 3. Limited access to clean water and personal hygiene products	1. Increased close contact facilitates Hepatitis B transmission. 2. Increased risk of infection through contaminated environments. 3. Facilitates indirect transmission via shared items like razors or toothbrushes.
Healthcare challenges	1. Inadequate healthcare infrastructure 2. Shortages of trained healthcare personnel 3. Lack of integration between prison health services and national healthcare systems	1. Insufficient access to diagnosis, vaccination, and antiviral treatments. 2. Limited capacity to manage Hepatitis B cases effectively. 3. Disruptions in continuity of care for inmates.
Cultural and social behaviors	1. Stigmatization of Hepatitis B 2. Lack of awareness about disease transmission among inmates 3. Cultural neglect of prisoner welfare	1. Deterrence from seeking testing and treatment. 2. Perpetuation of risky behaviors such as sharing of personal items and unsafe tattooing. 3. Reduced public and policy attention to prison health issues.
Risk behaviors in prisons	1. Use of unsterilized tattooing and piercing equipment 2. Sexual violence and unprotected consensual sexual activities 3. Drug use and sharing of needles	1. Direct transmission through blood contact. 2. Spread of the virus through bodily fluids. 3. High‐risk exposure to infected blood.
Policy and governance	1. Lack of targeted policies for Hepatitis B in correctional facilities 2. Weak enforcement of international prison health standards 3. Insufficient funding and resource allocation	1. Minimal screening, vaccination, and treatment initiatives for inmates. 2. Poor compliance with WHO and other global guidelines for Hepatitis B management. 3. Neglect of prison health issues in broader public health budgets.
Environmental and structural factors	1. Poor prison infrastructure 2. Limited access to educational programs for inmates and staff	1. Restricted access to isolation for infected individuals, increasing transmission risks. 2. Lack of awareness and prevention measures for Hepatitis B.

## 3 Innovative Approaches to Hepatitis B Virus Prevention Among African Prisons Inmates

A higher prevalence of HBV in prisons than in the general population has been reported in various studies [11, 25, 26]. In these settings, the highly contagious HBV can spread quickly among inmates and staff, leading to potential outbreaks both within and outside of prison walls. Preventive measures, such as education, vaccination, and screening, are cost‐effective and vital in protecting both inmates and staff from the severe consequences of HBV infections, which place additional strain on already stretched healthcare facilities. To effectively address HBV infection among this vulnerable population, a multifaceted approach that includes peer support and innovative technologies tailored to the unique socioeconomic and cultural contexts of these settings is necessary. This approach aligns with the Global Health Sector Strategy (GHSS) recommended by the World Health Assembly for eliminating hepatitis by 2030, highlighting the efficacy of preventive interventions and their potential for implementation [27]. The approaches involve:

I. **Education and awareness campaigns**: Literature has revealed poor knowledge of the transmission and prevention of HBV among prison inmates [11]. In a survey among inmates in a Geneva prison (with more than 25% of the inmates of sub‐Saharan origin), less than 30% had adequate knowledge of and protection against HBV transmission, and 72.2% of participants from sub‐Saharan Africa had chronic/resolved HBV infection [11]. Education programs targeting inmates and prison staff can play a crucial role in raising awareness about HBV transmission, prevention methods, and the importance of vaccination. Such educational initiatives, adapted to address literacy levels, cultural values, and language difficulties, are necessary to raise awareness about HBV among prisoners.
II. **Integration of HBV screening into prison health services**: The World Health Organization (WHO) recommends screening using serological tests to detect HBV surface antigen and other markers of infection in several high‐risk categories, including those incarcerated [28]. Using available point‐of‐care testing devices, screening can be part of prison health services to facilitate vaccination of those unaffected, identify infected individuals, provide prompt medical interventions, and implement preventive measures to mitigate further transmission [27]. In a study among prison entrants in Australia, HBV was detected in 20% of the sample; less than 30% had HBV vaccination coverage, and vaccination completion rates were suboptimal [29]. In a Liberian prison, a screening exercise revealed that 34% of HBV prevalence among participants with active infection was 14%, while 12.9% prevalence was recorded among Cameroonian prison inmates [5, 15]. These findings corroborate the fact that such comprehensive testing programs involving routine screening and point‐of‐entry screening for HBV would play a pivotal role in improving the care and welfare of inmates. They can break barriers to accessing healthcare services. By identifying individuals infected with HBV, these programs facilitate timely access to HBV vaccination and antiviral therapy by including vaccination initiatives, education campaigns, and linkage to care in prison health programs. This action can prevent disease progression and reduce the risk of transmission within prison populations and upon the release of inmates into their communities.
III. **Vaccination programs**: Vaccination against infectious illnesses is one of the most significant contributions to public health of the 20th century, saving millions of lives and billions of dollars every year [30]. Vaccination against HBV is recommended for those with HIV and chronic liver disease, including those with hepatitis C; thus, it is beneficial to susceptible individuals and the public if sufficient herd immunity is achieved [28]. For over two decades, vaccinations against HBV generated from recombinant DNA have been available, requiring three doses in the primary hepatitis B immunization sequence [28]. Vaccination campaigns within prisons have proven effective in preventing new HBV infections [26]. In Europe, national guidelines recommend that the HBV vaccine should be given to all individuals entering prison [26]. This decision aligns with the overarching disease prevention theory, which aims to minimize the spread of infection inside the prison environment [26]. Identifying susceptible individuals during intake activities or routine medical check‐ups can maximize coverage. Available evidence reveals that rapid vaccination within 2 months yields about 82.5% seroprotection for prison recipients [26]. Healthcare professionals and peer educators within the prison environment can be engaged in campaigns against HBV vaccine hesitancy. Vaccination services should be readily accessible within prisons by running special vaccination clinics or integrating vaccination into routine healthcare services. Additionally, community health providers should be engaged to ensure that vaccination regimens are maintained after release. Overall, implementing HBV vaccination programs within prisons is essential for preventing new infections and will contribute to the long‐term control of HBV [28].
IV. **Addressing stigma and promoting awareness**: Due to misunderstandings and false information on the transmission, prevention, and treatment of HBV, stigma can occur. In prison settings, where individuals are already marginalized, stigmatizing attitudes surrounding HBV perpetuate discrimination, exacerbating the burden of HBV. The fear of prejudice or unfavorable reactions from others may discourage other inmates from obtaining testing, seeking healthcare services, disclosing HBV status, or adhering to treatment regimens.

Peer‐led educational programs, delivered by qualified prisoners, provide a safe space for interaction and addressing misconceptions about the disease. These initiatives provided by peer educators who have received training in HBV management and advocacy skills provide dependable sources of knowledge and assistance. By fostering an understanding of transmission routes and prevention strategies, these programs empower inmates to take responsibility for their health and reduce the stigma associated with HBV. Across detention centers in Canada, inmates trained on infection prevention and control practices to prevent blood‐borne infections served as peer educators, providing peer education and counseling to other inmates with positive outcomes [31, 32].

Using peer networks reduces exposure to health risks, encourages positive health‐seeking behaviors, improves treatment adherence, and enhances the health and safety of staff, inmates, and the general public. Additionally, outreach sensitization campaigns can be conducted in collaboration with community organizations and local health authorities to raise awareness and reduce misconceptions about the virus. Integrating HBV awareness programs into health promotion activities is essential to reduce stigma and raise awareness about HBV. This can be achieved using various mediums, such as posters, leaflets, and interactive workshops, to effectively disseminate information and encourage adherence to preventive measures [27].

To conclude this section, comprehensive hepatitis prevention programs represent a critical intervention strategy for combating the HBV crisis in African prisons. By implementing innovative strategies tailored to the unique needs of incarcerated populations, significant progress can be made in preventing HBV transmission and improving the overall health outcomes of inmates. Understanding the socio‐cultural context and specific stigmatizing beliefs prevalent in African prisons is crucial for addressing structural, societal, and individual‐level factors associated with stigma. However, limited funding, inadequate infrastructure, political will, and a lack of trained healthcare personnel present significant obstacles. Furthermore, the integration of hepatitis services into the current healthcare infrastructure, addressing the stigma, discrimination, and abuses of human rights experienced by incarcerated individuals living with HBV, is essential to advancing fair access to healthcare and producing significant results.

### 3.1 Impact on Public Health Beyond Prison Walls

A. **Understanding the spillover effects of hbv in communities**
  HBV is a global health concern in the healthcare system, causing significant public health challenges. HBV transmission in prison settings has a growing spillover effect beyond prison walls, impacting not only the inmates but their families, visitors, and broader communities. Inmates within prison facilities usually live in unsanitary conditions and are prone to contracting and transmitting infectious diseases [21].
  Policy reforms and efforts on advocacy are necessary for HBV Vaccination in prison facilities across Africa to address spillover effects of local communities. Collaborative efforts are needed between public health agencies, community organizations, and prison authorities to facilitate resource information sharing and joint action to tackle this disease agent. They recognize the interconnectedness between public health outcomes and Hepatitis B infection in prisons. This emphasizes the importance of collaboration in building a healthy and more resilient community [33, 34].
B. **Increased risks for family members and visitors**
  One of the significant means by which HBV is transmitted across prisons in Africa is through contact between inmates and their family members or visitors. Several factors contribute to this heightened risk [28, 35, 36].
  1. **Limited awareness and screening**: Most family members are unaware of HBV transmission risks and prevention. Usually, they are unvaccinated or unscreened in the first place against HBV, and this will increase their susceptibility to transmission.
  2. **Inadequate infection control measures**: Most African prison facilities lack effective control measures to prevent HBV transmission during visit hours. There are limited clean facilities and other cleaning protocols, which in turn facilitate the spread or spillover effects of HBV among visitors and prisons.
  3. **Stigma and discrimination**: The accompanying stigma and discrimination of HBV patients often may deter other individuals from disclosing their health status to healthcare services or prison facilities and pose a threat to other inmates, visitors, and family members

C. **Broader implications for public health systems**
  The spillover effects of HBV from prison settings to communities have broader implications for public health systems, which include [37]:
  1. **Increased healthcare burden**: The transmission of HBV from inmates to families and visitors and, by extension, the general public will place an additional strain on healthcare systems in Africa, leading to increased demand for HBV Screening, vaccination, and treatment protocols. This increase in burden on healthcare facilities can overwhelm the existing resource‐constrained healthcare settings.
  2. **Challenges in contact tracing**: Tracking HBV‐infected individuals within communities poses significant threats for public health agencies. Due to inadequate surveillance systems, strain on resources, and other factors, suboptimal control of the HBV transmission chain lingers, which threatens public health and safety [38].

The HBV challenges in African prisons go beyond inmate health to broader public health. Addressing HBV challenges in prisons is, therefore, critical for protecting citizens from the spread of infectious diseases. Effective Hepatitis B Vaccination in African prisons requires a stakeholder approach involving healthcare providers and prison authorities to leverage expertize toward a common goal. Engaging with local communities and the inmates is pivotal as it ensures the interventions are culturally appropriate, sustainable, and responsive [39].

### 3.2 Challenges and Opportunities for Future Directions and Intervention

One of the biggest challenges in combating hepatitis B in Nigerian prisons is the lack of education and awareness about the disease among inmates and prison staff, as most individuals infected with HBV are unaware of their infection. This situation is exacerbated by the lack of screening programs and proper health services in prisons; as a result, many cases of hepatitis B go undetected, making treatment difficult and facilitating the spread of the disease among the prison population [40, 41].

Nigerian prisons are often overcrowded due to pretrial detention and delayed trials of inmates. Such crowded environments promote the transmission of hepatitis B among inmates [42, 43]. Factors such as poor access to health care among inmates, including shortage of medical personnel and diagnostic and treatment facilities, often limit effective diagnosis and treatment of hepatitis B cases in prisons [42, 44]. Furthermore, the lack of understanding of the disease and fear of how it is transmitted often lead to stigmatization and discrimination among those incarcerated; as a result, affected individuals often refrain from seeking medical care [11].

These challenges can be addressed through prison‐based HBV prevention strategies involving prison personnel, administration, and government. This includes the implementation of education and awareness campaigns on Hepatitis B in prisons to increase the understanding of the disease, reduce stigma, and encourage early testing to facilitate effective treatment and limit the spread of the disease among inmates and staff. Policies that prioritize Hepatitis B vaccination and access to regular screening and therapy for the incarcerated should be introduced and appropriately implemented by the government to reduce the incidence of the infection in prisons and the general population after inmates are released. High‐risk practices such as injecting drug use, unprotected sex, body piercing, and tattooing should be strongly discouraged [3, 45, 46].

Inmates and staff should be encouraged to practice good personal hygiene to limit the spread of HBV [47]. The government should invest more in health infrastructure and the provision of health services in Nigerian prisons, as the current health service in most prisons is inadequate to combat Hepatitis B effectively [44]. In addition, the prison service could work with nongovernmental organizations, external health organizations, and government agencies to provide resources to support hepatitis B interventions in Nigerian prisons.

Furthermore, prisoners should have equal access to healthcare in prisons, with the same quality of health services as the general population. This should include equitable distribution of healthcare resources, medical personnel, treatment facilities, and diagnostic tools across prisons in Nigeria [48]. Monitoring and evaluation mechanisms by authorized bodies should be implemented to track the provision of health services within prison settings and identify gaps in access to quality hepatitis B treatment.

## 4 Conclusion

In conclusion, the study highlights the severe health risks faced by prisoners in Africa, further worsened by overcrowded and unsanitary conditions, limited access to healthcare, and insufficient screening and vaccination programs. Addressing Hepatitis B in African prisons is not only a matter of improving prisoner health but also a critical public health strategy to prevent the spread of the virus outside prison walls. Implementing these measures will require political will, community support, and sustained investment, but the potential benefits to public health and social justice make it important. Overall, the study emphasizes that tackling Hepatitis B in African prisons is a pressing public health emergency that demands immediate and concerted efforts to protect vulnerable populations and enhance public health outcomes across the continent.

## Author Contributions

**Godfred Yawson Scott:** conceptualization, investigation, supervision, writing – original draft, project administration, writing – review and editing. **Abdullahi Tunde Aborode:** conceptualization, investigation, supervision, writing – original draft, writing – review and editing. **Ridwan Olamilekan Adesola:** conceptualization, project administration, supervision, writing – original draft, writing – review and editing. **Chime Onyinye Hope:** writing – original draft, writing – review and editing. **Olayide Dipeolu:** writing – original draft, writing – review and editing. **Favour Akinfemi Ajibade:** writing – original draft, writing – review and editing. **Abraham Olutumininu Akiyode:** writing – original draft, writing – review and editing. **Stephen Tetteh Engmann:** writing – original draft, writing – review and editing. **Abdullahi Jamiu:** writing – original draft, writing – review and editing. **Awoyemi Praise‐God Adetunji:** writing – original draft, writing – review and editing. **Joseph Agyapong:** writing – original draft, writing – review and editing. **Toluwalope Yinka Oni:** writing – review and editing, writing – original draft. **Mpanga Derick Denis:** writing – original draft, writing – review and editing. **Isreal Onifade Ayobami:** writing – original draft, writing – review and editing. **Adetolase Azizat Bakre:** writing – review and editing, writing – original draft.

## Ethics Statement

The authors have nothing to report.

## Conflicts of Interest

The authors declare no conflicts of interest.

## Transparency Statement

The lead author Godfred Yawson Scott affirms that this manuscript is an honest, accurate, and transparent account of the study being reported; that no important aspects of the study have been omitted; and that any discrepancies from the study as planned (and, if relevant, registered) have been explained.

## Data Availability

The corresponding author has the right to share the data available in the manuscript.
